# The Role of Neurohypophysial Hormones in the Endocrine and Paracrine Control of Gametogenesis in Fish

**DOI:** 10.3390/cells14141061

**Published:** 2025-07-10

**Authors:** Maya Zanardini, Hamid R. Habibi

**Affiliations:** Department of Biological Sciences, University of Calgary, 2500 University Drive NW, Calgary, AB T2N 1N4, Canada; maya.zanardini@ucalgary.ca

**Keywords:** paracrine regulation, steroidogenesis, oogenesis, spermatogenesis, vasotocin, isotocin, fish endocrinology

## Abstract

Arginine vasopressin (AVP) and oxytocin (OXT) are neuropeptides traditionally recognized for their roles in the control of osmoregulation, blood pressure, lactation, and parturition in mammals. However, growing evidence suggests that AVPand OXT also regulate gonadal functions in teleost fish. Their expression in both male and female gonads, the presence of their receptors in ovaries and testes, and their interactions with steroids and other gonadal factors indicate a role in modulating gametogenesis and steroidogenesis via autocrine and paracrine mechanisms. Here, we review the current findings on AVP and OXT in teleost gonads, compared to the observed functions in mammals, emphasizing their systemic interactions within the hypothalamic–pituitary–gonadal (HPG) axis. While highlighting the roles of gonadal AVP and OXT in fish reproduction, we underscore the need for further research to unravel their complex multifactorial regulatory networks. Insights into the vasopressinergic system could enhance aquaculture practices by improving spawning success and reproductive efficiency.

## 1. Introduction

Arginine vasopressin (AVP) and oxytocin (OXT) are neuropeptides primarily recognized for their classical roles in mammals, where AVP regulates osmotic balance and blood pressure and OXT regulates labor and milk ejection. At the same time, OXT is essential for lactation and parturition. However, growing evidence suggests that these peptides play additional roles in the control of reproduction. In fish, the vasopressin/oxytocin system has been extensively studied in the context of reproductive and social behaviors [1,2,3,4,5]. Yet, their roles at the gonadal level remain less explored. AVP and OXT have been identified in the gonads of both male and female fish [6,7,8], and they are known to interact with steroids and other gonadal factors [9], suggesting that these peptides are also produced peripherally and carry out locally mediated functions in the gonads. Recent studies have further highlighted their autocrine and paracrine roles at the level of the gonads, indicating a direct involvement in the key reproductive processes, including gametogenesis and steroidogenesis [8,10,11,12,13,14].

This review aims to provide a comprehensive analysis of the local regulatory functions of AVP and OXT within the gonads of teleost fish. We begin by outlining the fundamental hormonal mechanisms regulating spermatogenesis and oogenesis, emphasizing the role of gonadal nonapeptides in these processes. We then compare the reproductive roles of these neuropeptides in mammals and teleosts to highlight differences and similarities. Finally, we discuss the systemic interactions of AVP and oxt within the hypothalamic–pituitary–gonadal axis, highlighting the emerging evidence of their direct and indirect autocrine/paracrine regulation of gametogenesis and the overall gonadal function.

## 2. The Hypothalamic–Pituitary–Gonadal Axis

The hypothalamic–pituitary–gonadal (HPG) axis is the primary regulatory system responsible for gamete production, including oocytes and spermatozoa. In response to external and internal stimuli, the hypothalamus secretes several factors that function as upstream regulators by activating or inhibiting downstream signaling pathways [15]. Stimuli are first detected by a subpopulation of kisspeptin neurons in the arcuate hypothalamic nucleus that is co-expressed with neurokinin-B (NKB) and dynorphin-A, commonly referred to as the KNDy neurons, which modulate the synthesis and pulsatile release of the gonadotropin-releasing hormone (GnRH) by neurons located in the midbrain and hypothalamus in mammals [16,17,18,19,20,21]. GnRH, in turn, stimulates gonadotrophs in the anterior pituitary to secrete gonadotropins, the follicle-stimulating hormone (FSH), and the luteinizing hormone (LH). Gonadotropins enter the bloodstream and, upon reaching the gonads, regulate gonadal functions, including the synthesis of sex steroids and gonadal factors, ultimately leading to gamete production. While in mammals, GnRH neurons project into the median eminence and GnRH is released into the hypothalamic–pituitary portal system in a pulsatile fashion [22,23], teleost fish lack the median eminence, and the hypothalamic neurons directly project onto or in the vicinity of target pituitary cells [22,24]. While GnRH and kisspeptins are required for mammalian reproduction [25], this is not the case in fish. Studies in zebrafish showed that single or double knockouts of *gnrh2/gnrh3* and *kiss1/kiss2* and their receptors do not significantly impair gonadal development, and even triple knockouts of *gnrh3*, *kiss1*, and *kiss2* allow for physiological development and reproduction [25,26,27,28]. These findings suggest that compensatory mechanisms exist, indicating that the GnRH–kisspeptin system may be non-essential for reproduction in teleost fish [28]. Recently, the neuropeptide secretoneurin (SN) has drawn attention as a possible regulator of reproduction, as it stimulates LH release in goldfish and mouse models in a GnRH-independent way and may serve as an upstream regulator of the kisspeptin and GnRH systems [29,30,31]. The role of the hypothalamic–pituitary complex in modulating reproduction across vertebrates is critical, and a comprehensive set of information on the many factors and hormones involved in this control has been extensively covered [24,32,33].

### 2.1. Gonadal Steroidogenesis

In vertebrates, including fish, gonads are responsible for both gametogenesis and steroidogenesis, two integrated processes operating simultaneously to ensure reproductive success. Steroid hormones produced in the gonads regulate various aspects of reproduction, including mating behaviors, gamete production and development, and other reproductive functions through endocrine signaling. Steroid biosynthesis in fish takes place mainly in the gonad, head kidney, and brain, but the liver, gills, and adipose tissue are also important sites affecting steroid levels [34]. Gonads and inter-renal cells of the head kidney mainly produce sex steroids (androgens, estrogens, and progestins) and corticosteroids (glucocorticoids and mineralocorticoids), respectively [35].

In gonads, steroidogenesis occurs in testicular Leydig cells in males and ovarian theca and granulosa cells in females. Steroid hormones are all synthesized from cholesterol, which is transported from the outer to the inner mitochondrial membrane by the steroidogenic acute regulatory protein (StAR). This transport is considered the rate-limiting step of steroidogenesis, and the physiological function of the StAR protein and the *star* gene has been extensively studied using gene editing, including single- and double-knockout fish. Two duplicated *star* genes, namely *star1* and *star2*, have been identified in non-mammalian vertebrates, including fish. *Star1* is mainly expressed in the ovaries, testes, and head kidney, while *star2* is specific to the gonads [36]. Despite the importance of StAR, zebrafish with *star1* knockout showed no effects on testicular differentiation or spermatogenesis. However, their testosterone levels were significantly elevated [36]. In Nile tilapia (*Oreochromis niloticus*), deficiency of the *star2* gene caused delayed spermatogenesis, sperm apoptosis, and sterility. Similarly, in double knockout tilapia (*star1^−/−^*/*star2^−/−^*), spermiogenesis was impaired, and no mature sperm were produced. *Star1* deficiency resulted in abnormal sperm morphology, reduced motility, and decreased fertility; however, fertility was restored upon supplementation with 11-ketotestosterone (11-KT) or cortisol in the *star1^−/−^* mutants [37]. Overall, these findings suggest that the two *star* genes in fish may work together to initiate steroidogenesis and support male fertility. Other genes encoding three steroidogenic enzymes, *cyp11a1*, *cyp17*, and *cyp19a1a*, play a crucial role in regulating steroidogenesis. The cytochrome P450 side-chain cleavage enzyme, encoded by the *cyp11a1* gene, catalyzes the first step of steroidogenesis, converting cholesterol to pregnenolone by reducing the number of carbons from C27 to C21 [38]. Next, pregnenolone undergoes sequential enzymatic conversion into key active steroids in fish, including 17α,20β-dihydroxy-4-pregnen-3-one (DHP), testosterone (T), 11-KT, estradiol-17β (E2), and the corticosteroids. CYP17A is crucial for converting C21 to C19 steroids, producing 17α-hydroxyprogesterone and androstenedione. CYP17A exhibits dual enzymatic activity, functioning as both a 17α-hydroxylase and a 17,20-lyase [39].

Granulosa cells express the enzyme aromatase, a key enzyme that converts androgens provided by theca cells into estrogens. Aromatase is conserved in fish, but teleosts uniquely possess two isoforms with distinct tissue distributions: *cyp19a1a*, expressed in the gonads, and *cyp19a1b*, primarily found in the brain [40,41]. *Cyp19a1a* plays a crucial role in gonadal sex differentiation and gametogenesis, whereas *Cyp19a1b* is involved in brain cell proliferation and neurogenesis, thereby supporting brain development and repair [40,42].

Functionally, the main sex steroids produced in the gonads—androgens, estrogens, and progestins—exert coordinated roles in regulating gametogenesis. 11-KT is a potent male-specific androgen in teleosts and has been shown to play a key role in regulating testicular gene expression and promoting spermatogenesis in several species [43,44,45,46]. While androgens are indispensable for spermatogenesis in mammals, teleost fish exhibit species-specific degrees of androgen dependency. For instance, cyp11a2−/− mutant zebrafish exhibit impaired breeding behavior and sperm maturation, with upregulation of spermatogonial markers (nanos2, piwil1) and downregulation of meiotic and spermatid markers (sycp3, odf3b/cimap1b), indicating normal differentiation of early spermatogonia with compromised transition to/or completion of meiosis [47]. Similarly, cyp17a1−/− mutants exhibit reduced testosterone and 11-KT levels, poor sperm quality, and decreased mating behavior, while spermatogenesis remains unimpeded [48].

Androgens act via androgen receptors (ARs) expressed in Sertoli and interstitial cells in the testes. Androgens also play a key role in ovarian development, with ARs being expressed in theca and granulosa cells surrounding follicles in the vitellogenic and post-vitellogenic stages in the ovaries [49]. In coho salmon, 11-KT exposure during the perinucleolar stage accelerated follicle growth and altered the transcriptome of early secondary oocytes, affecting genes involved in gonadotropin signaling, steroidogenesis, and lipid uptake [50,51]. The authors suggested that 11-KT may modify the follicle’s ability to produce and respond to sex steroids and, in accordance with studies in other species, may modulate lipid (and vitellogenin) incorporation in ovarian follicles [50,51]. Kortner et al. (2009) observed similar results in immature female Atlantic cod [52]. There, both 11-KT and T were capable of inducing oocyte growth, but exposure to 11-KT produced the strongest modulation [52]. Loss of AR in male zebrafish disrupts but does not block spermatogenesis. Mutants exhibited reduced testis weight and fewer functional spermatozoa, likely due to decreased levels of steroid hormones, including E2 and 11-KT. Despite this, they were infertile due to an inability to release sperm [53]. These findings emphasize that androgens are crucial but not solely sufficient for full reproductive competence. This suggests that other factors—including autocrine and paracrine signaling mechanisms—may partially compensate to support gametogenesis in an androgen-independent manner. This topic will be further explored below.

Estrogens regulate oogenesis, vitellogenesis, gonadotropin control, testicular development, and other reproductive and systemic functions [54,55,56,57]. During ovarian development, plasma E2 peaks at vitellogenesis, stimulating hepatic vitellogenin production and oocyte growth. E2 biosynthesis is primarily modulated by FSH and, to a lesser extent, LH, inducing expression of steroidogenic enzymes, including P450 aromatase (*cyp19a*) in ovarian granulosa cells [58,59]. Three estrogen nuclear receptors (ESR1, ESR2A, and ESR2B) and one estrogen membrane receptor (GPER) have been identified in zebrafish [60]. *Esr1* and *esr2b* were expressed in the follicular cell layer, whereas *esr2a* was present in both the follicular layer and oocytes. Conversely, the membrane receptor *gper* mRNA and protein were mainly found in the oocytes [61]. Single knockout of either of the nuclear estrogen receptors did not affect fertility; however, double or triple knockouts had follicles arrested in the previtellogenic stage and exhibited a shift towards a testis-like structure, especially in *esr2a* and *esr2b* mutants, indicating that at least these two receptors are required to mediate the role of estrogen in sex determination [25]. GPER knockout had minimal effect on ovarian development, suggesting potential compensatory mechanisms via another membrane or nuclear estrogen receptors [25,62,63].

The role of E2 in testis development was first hypothesized by Miura et al. in 1999, who observed that E2 induced mitosis in spermatogonial stem cells from cultured testicular tissue of Japanese eel (*Anguilla japonica*) [64]. A few years later, Bouma et al. (2001) provided the first evidence of ER-α protein localization in the interstitial compartment and blood vessels of the rainbow trout (*Oncorhynchus mykiss*) testis [65]. On the other hand, studies on zebrafish *cyp19a1a* and *cyp19a1b* single and double-mutant lines revealed that male fish lacking aromatase maintained normal fertility even at one-year post-fertilization [66]. These mutants had higher sperm counts compared to wild-type and *cyp19a1b* mutants, along with increased androgen levels, elevated FSHβ and LHβ expression in the pituitary gland, and upregulated testicular genes involved in spermatogenesis and steroidogenesis [66]. The functional role of estrogens in fish spermatogenesis appears to be species-specific. E2 appears to be responsible for modulating early spermatogonial proliferation, but it is not required for completing spermatogenesis, as evidenced by the enhanced activity of androgens and gonadotropins that may compensate for the loss of estrogen signaling.

Progestins are steroid hormones derived from progesterone, commonly referred to as maturation-inducing steroids (MIS) due to their role in stimulating resumption of meiosis, oocyte germinal vesicle breakdown (GVBD), final oocyte maturation, and ovulation in females [59,67,68]. In salmonids and many other fish species, DHP is the primary active MIS, stimulated by LH and acting through membrane progestin receptors to activate the maturation-promoting factor, leading to oocyte maturation [58,69]. Three progesterone receptors have been identified in zebrafish: the nuclear progestin receptor (nPGR) and two membrane progestin receptors, mPGRα and mPGRβ. The nPGR is particularly important in ovulation, with its expression peaking at the full-grown follicle stage and being directly stimulated by LH, as demonstrated in zebrafish both in vivo and in vitro [70]. Interestingly, female zebrafish deficient in the *npgr* gene exhibited normal oocyte maturation but were infertile due to a failure to ovulate [70,71].

In males, DHP also plays a significant role in spermatogenesis. Male zebrafish treated with DHP showed increased proliferation and differentiation of early spermatogonia, as well as the induction of meiosis in spermatocytes [72]. DHP has also been implicated in sperm maturation and the acquisition of motility, although the underlying mechanisms remain unclear [73]. In male cyprinids, DHP functions as a pheromone; its release by female goldfish during final oocyte maturation triggers sperm release and induces courtship behavior in males [74]. Studies in tilapia further highlight the role of progesterone signaling in male fertility. Knockout of *npgr* in male tilapia resulted in disorganized spermatogenic cysts, reduced sperm count and motility, and a decrease in spermatocytes and spermatozoa, indicating that nPGR is crucial for male fertility in this species [75]. Notably, DHP administration in *ar^−/−^* knockout male zebrafish effectively restored the phenotype, gonadosomatic index (GSI), and spermatozoa numbers to those of wild-type fish. Furthermore, while *cyp17a1^−/−^*; *ar^−/−^* double-knockout fish exhibited hypertrophic testes and enhanced spermatogenesis, triple-knockout (*cyp17a1^−/−^*; *ar^−/−^*; *npgr^−/−^*) fish showed defective spermatogenesis and disorganized testes. This suggests that accumulated progestins (DHP and progesterone, resulting from *cyp17a1* depletion) may play an alternative signaling role in promoting testis organization and spermatogenesis independent of androgen signaling [76].

### 2.2. Gametogenesis

#### 2.2.1. Oogenesis: An Overview with a Focus on Paracrine/Autocrine Factors

Oogenesis is the process by which oogonia develop into mature eggs, known as ova. Each stage of development is tightly regulated by endocrine signals, with E2 and DHP being among the key hormones that stimulate oogonial proliferation and the subsequent entry into meiosis [59]. The ovarian follicle is the functional unit of female gametogenesis, which consists of an oocyte surrounded by a monolayer of granulosa cells and an outer layer of theca cells. While the oocyte remains arrested in prophase I, the follicle undergoes three developmental stages: primary growth, secondary growth, and final oocyte maturation. During the primary growth, the oocyte associates with follicular cells (theca and granulosa) and undergoes intense transcriptional activity in preparation for later stages. The secondary growth is marked by vitellogenesis—the synthesis and accumulation of yolk proteins. This process is modulated by pituitary FSH, which promotes P450 aromatase activity essential for E2 synthesis that directly regulates hepatic vitellogenin production [59]. Vitellogenin is then cleaved into smaller yolk proteins by the lysosomal enzyme cathepsin D, providing a nutrient-rich reserve of proteins, lipids, and vitamins necessary for embryogenesis and larval development [59,77]. Paracrine and autocrine factors produced within the ovary contribute to follicle development and oocyte maturation by partially regulating gonadotropin activity (as shown below). For example, growth differentiation factor 9 (GDF9) is an oocyte-secreted factor essential for early follicle development in mammals. In zebrafish, *gdf9* transcript is highly expressed during the primary growth stage, but its level decreases as follicle development progresses, suggesting it may be important for the development of oogonia and primary follicles [78]. Similarly, the anti-Müllerian hormone (AMH) is expressed in the follicular granulosa cells during primary and secondary growth but declines in pre-vitellogenic follicles [79]. The expression pattern of *amh* is opposite to that of aromatase (*cyp19a*), with the absence of *amh* expression coinciding with maximal expression of aromatase, suggesting that AMH may suppress the expression of aromatase and, in turn, inhibit vitellogenesis [79].

In post-vitellogenic oocytes, the LH surge induces a shift in the steroidogenic pathway from E2 toward the production of DHP, recognized as the primary MIS across a variety of fish species [68,80]. MIS binds to membrane progestin receptors, activating the maturation-promoting factor (MPF), a complex of Cdc2 kinase and cyclin B, which triggers GVBD, resumption of meiosis, and ovulation [69].

There is evidence that bone morphogenetic protein 15 (BMP15), expressed throughout follicle development, may play a role in follicle secondary growth and maturation. BMP15 antibody injection into zebrafish oocytes reduced the number of vitellogenic follicles while increasing the number of mature follicles. Additionally, the overexpression of zebrafish *bmp15* in oocytes inhibited human chorionic gonadotropin (hCG)-induced oocyte maturation [81]. These findings together suggest that BMP15 may promote follicle growth while preventing premature maturation [82].

Other factors belonging to the transforming growth factor beta (TGF-β) superfamily, including activin and epidermal growth factor (EGF), are involved in paracrine regulation of ovarian development. Activin subunits (βA and βB) and follistatin, an activin-binding protein, are the key regulators of oocyte maturation, functioning as part of the ovarian activin–follistatin system to mediate gonadotropin signaling [83]. Gonadotropins regulate the expression of both activin and follistatin in cultured zebrafish follicle cells, highlighting their role in follicular development. During vitellogenesis, the activin βA subunit increases, suggesting its involvement in promoting follicle growth. Activin also plays a crucial role in stimulating LH-induced oocyte maturation, while follistatin counteracts this effect by inhibiting hCG-induced maturation [83,84]. EGF has also been found to mediate the effect of LH in oocyte maturation by acting through activin. EGF stimulates the expression of activin and inhibits follistatin expression independently from hCG [85].

After maturation, the oocyte is arrested again at metaphase II until fertilization. Right up to ovulation, the activation of aquaporins facilitates oocyte hydration, a crucial process that maintains egg viability, particularly in marine species [59,86]. The effects of LH on ovulation are mediated by various factors secreted by follicular cells, including proteases, prostaglandins (PGs), and insulin-like growth factors (IGFs). Members of the insulin-like growth factor family (IGF1, IGF2, and IGF3) are expressed in early oocytes and post-vitellogenic follicular cells and seem to play an essential role in the survival of granulosa and theca cells, oocyte maturation, and ovulation [87,88]. IGF3 has been identified as a crucial mediator of LH-induced oocyte maturation in zebrafish, both in vivo and in vitro [89]. GnRH is also produced within the ovaries of zebrafish and goldfish, playing a regulatory role in the final stages of oocyte maturation, stimulating GVBD and ovulation [90,91]. In zebrafish, both GnRH3 and the gonadotropin-inhibitory hormone (GnIH) are expressed in pre-, mid-, and late-vitellogenic oocytes, and both show a direct action on oocyte maturation. In particular, GnIH appears to play a crucial role in regulating the final oocyte maturation in the presence of GnRH and gonadotropins [90]. These findings suggest a complex regulatory interplay between ovarian GnRH and GnIH, highlighting their potential coordinated roles in controlling the final oocyte maturation and ovulation.

#### 2.2.2. Spermatogenesis: An Overview with a Focus on Paracrine/Autocrine Factors

Spermatogenesis is the process of sperm cell development, during which germinal cells undergo three main stages: the mitotic phase, the meiotic phase, and spermiogenesis. Before exploring some of the key factors and mechanisms that regulate spermatogenesis, it is essential to note that FSH and LH play complementary roles in this process, with FSH controlling the early stages and LH overseeing the final maturation steps. Different steroids—progestogens, androgens, cortisol, and estrogens—play stage-specific roles. Low levels of E2 promote self-renewal of spermatogonia, while androgens initiate spermatogonial proliferation, differentiation, and meiosis. Cortisol supports proliferation and meiotic entry, whereas DHP is crucial for both meiosis initiation and spermiation [74,92,93,94,95,96]. The mitotic phase begins with a spermatogonial stem cell (SSC) localized in a specific region known as the testicular “niche” [94,97,98]. The niche is formed by Sertoli cells, Leydig cells, peritubular myoid cells, and endothelial cells and offers a specific microenvironment that allows SSCs to maintain their “stemness” characteristics, including self-renewal, pluripotency, and quiescence [99,100,101]. Once SSCs commit to differentiation, they develop into type A undifferentiated (Aund) spermatogonia, followed by type A differentiated (Adiff) spermatogonia, type B spermatogonia (SpgB), and then spermatocytes (Spc). Spermatocytes undergo two rounds of meiosis, resulting in spermatids, which subsequently mature into spermatozoa. Self-renewal of SSCs and early spermatogonia type A proliferation and differentiation in zebrafish are influenced by systemic and local pathways. In the Japanese eel, low concentrations of E2 are required to maintain the proliferation of spermatogonia type Aund [64].

In addition to estrogens, pituitary FSH plays a crucial role in modulating paracrine and autocrine regulatory pathways in both an androgen-dependent and -independent manner. While mammals show a distinct expression of FSH and LH receptors—LH receptors in Leydig cells stimulating steroidogenesis, and FSH receptors in Sertoli cells regulating cell development—in fish, FSH receptors are present in both Leydig and Sertoli cells, and FSH acts as a potent steroidogenic hormone [102]. FSH also modulates the key signaling pathways involved in regulating germ cells. Locally produced factors that influence gametogenesis in the testes are summarized and illustrated below. One such pathway is the WNT signaling cascade, where WNT5A, a glycoprotein produced by Leydig cells, plays a crucial role in the self-renewal of germ cells. The evolutionarily well-conserved WNT signaling pathway influences essential cellular processes, such as fate determination, migration, and renewal, during early vertebrate development [103]. In zebrafish, FSH regulates the *wnt5a* expression, which promotes the self-renewal of type Aund spermatogonia, supports their proliferation and accumulation, and stimulates the formation of new Sertoli cells in an androgen-independent manner [104,105].

Studies have shown that germ cell-derived factors, such as glial cell line-derived neurotrophic factor (GDNF), also regulate self-renewal in an autocrine manner. *Gdnf* and its receptor *gfrα1* (gdnf family receptor α1) are expressed in type A undifferentiated spermatogonia in many fish species, with their expression gradually decreasing during germ cell development [106,107]. Doretto et al. (2022) demonstrated that recombinant human GDNF promotes spermatogonial proliferation while inhibiting their differentiation into type B spermatogonia in zebrafish testes [108]. Additionally, similarly to WNT5A, GDNF induces Sertoli cell proliferation, contributing to the formation of a new niche [108].

FSH regulates the production of two opposing Sertoli-derived growth factors, IGF3 and AMH, both of which belong to the TGF-β family [109,110]. IGF3 is a teleost gonad-specific growth factor that, in zebrafish, stimulates the proliferation and differentiation of Aund and Adiff spermatogonia in an androgen-independent manner and also upregulates the expression of testicular transcripts related to spermatogonial differentiation [110,111]. On the other hand, in adult zebrafish, recombinant AMH was found to exert the opposite function, i.e., inhibiting androgen-induced proliferation and differentiation of type A spermatogonia, blocking these cells from differentiating [112]. In the European sea bass (*Dicentrachus labrax*), *amh* expression in Sertoli cells decreases at the onset of puberty, with FSH administration promoting spermatogonial proliferation and reducing *amh* mRNA levels [113]. Similarly, in prepubertal Japanese eel, AMH inhibits gonadotropin- or androgen-stimulated spermatogenesis [92]. The regulation of AMH is species-dependent, with its expression being downregulated by FSH or androgens [92,112]. Importantly, AMH plays a key role in modulating IGF3 production in Sertoli cells by inhibiting FSH-induced *igf3* expression, thereby counterbalancing its proliferative and differentiative effects. In addition, AMH promotes the release of inhibitory factors such as inhibin-A and prostaglandin E2 (PGE_2_), both of which suppress the proliferation and differentiation of type A spermatogonia [114,115]. This finely tuned regulatory network ensures the maintenance of a germ cell pool while preventing excessive or uncontrolled progression of spermatogenesis.

Under the influence of FSH, Leydig cells produce insulin-like peptide 3 (INSL3) and androgens [116]. In zebrafish, the *insl3* transcript level increased significantly after FSH treatment but not in response to LH. More recently, INSL3 receptors *rxfp2a* (relaxin family peptide receptor 2a) and *rxfp2b* were found to be expressed by type A spermatogonia and Sertoli and myoid cells, respectively [117]. Testes treated with human and zebrafish INSL3 showed increased differentiation and proliferation of type Aund spermatogonia to type Adiff without stimulating androgen production [116,117]. These cells then proliferate, reduce in size, and are classified as type B spermatogonia. Once again, FSH plays a crucial role in stimulating Sertoli cells’ production of IGF3, inducing proliferation towards type B spermatogonia. These cells undergo rapid proliferation, with the number of proliferative cycles being species-specific and varying remarkably among fish. For instance, zebrafish exhibit 9 spermatogonial generations, while mosquitofish (*Gambusia affinis*) have 10–12, and tilapia have 7 [96,118]. Cell development continues to produce spermatocytes, smaller cells that characterize the meiotic stage, as they undergo meiotic divisions to produce spermatids—small, haploid cells with a round nucleus. The final phase, spermiogenesis, transforms spermatids into mature spermatozoa through extensive morphological changes without further cell division. This process includes nuclear condensation, elimination of organelles and the cytoplasm, and the formation of a flagellum. The release of mature spermatozoa into the lumen of the seminiferous tubule occurs due to the breakdown of intercellular bridges and reorganization of Sertoli cells [96].

Retinoic acid (RA) is another factor that plays a crucial role in regulating zebrafish spermatogenesis. Produced in the testes under FSH regulation, RA promotes spermatogonial differentiation, reduces germ cell apoptosis, and modulates the testicular function [119]. Its effects complement those of 11-KT, though the two hormones have distinct roles. RA stimulates the transition from Aund to Adiff and type B spermatogonia, inducing this stage-specific proliferation while also reducing apoptosis in spermatids [119]. Thus, RA primarily supports spermiogenesis, thereby increasing its efficiency, whereas 11-KT is essential for completing meiosis. Interestingly, a reciprocal regulatory mechanism exists between RA and androgens. While androgen signaling supports RA production, RA, in turn, reduces androgen synthesis by inhibiting Leydig cell function, potentially serving as a mechanism to prevent testicular hypertrophy and maintain testicular homeostasis [119].

Another important local regulator of spermatogenesis is GnRH. Studies have shown that GnRH isoforms (*gnrh2* and *gnrh3*) and their receptors (*gnrhr1*, *gnrhr2*, *gnrhr3*, *gnrhr4*) are expressed in the testes of zebrafish and goldfish [120,121]. Interestingly, in zebrafish testes, *gnrh3* expression is 3–4 times higher than that of *gnrh2*, though GnRH2 appears to be the most potent form. Both GnRH isoforms were found to increase the mitotic activity of type Aund spermatogonia, promoting sperm formation in zebrafish testes in an androgen-dependent manner. Additionally, they inhibited FSH-induced spermatogenesis, though their effect on hCG-induced spermatogenesis is less pronounced [121]. In seasonal breeders such as goldfish, the effects of GnRH peptides were found to vary with the stage of testis development. For instance, during early and mid-maturation, both GnRH isoforms had a protective role or no effect, whereas in fully mature testes, both peptides induced apoptosis [122]. Similarly, GnIH was also found to be involved in spermatogenesis as a local regulator that fine-tunes the spermatogenic process in response to hormonal and local signals. In goldfish, the expression of GnIH receptor (*gnihr1* and *gnihr2*) mRNA has been demonstrated in Leydig cells [123]. More recently, the expression of GnIH in both germ cells and Leydig cells was confirmed in zebrafish testes [124]. In zebrafish, GnIH exhibits complex, often dose-dependent effects on both basal spermatogenesis and gonadotropin-induced responses [124,125]. It was demonstrated that a lower dose of GnIH (10 nM) inhibited gonadotropin-induced spermatogenesis by blocking FSH- and hCG-induced androgen production in testes treated ex vivo. Additionally, the testes exhibited a reduction in spermatids and spermatozoon content, as well as decreased mitotic activity of type A differentiated spermatogonia while increasing the proliferation of type B spermatogonia. On the other hand, the highest dose tested (1000 nM) increased basal numbers of haploid cells as well as testosterone production, exhibiting a “paradoxical effect” [124,125].

In addition to locally produced factors, thyroid hormones also play a significant role in the multifactorial regulation of fish spermatogenesis. Strong evidence suggests that triiodothyronine (T3) plays a crucial role in priming germ cells for meiosis and influencing post-meiotic stages [126]. T3 treatment in zebrafish testes has been shown to stimulate the proliferation of Sertoli cells and type Aund spermatogonia while also increasing the mitotic activity of type A and B spermatogonia, spermatocytes, and spermatids. Additionally, T3 increases FSH-induced 11-KT production and basal *igf3* expression, highlighting its role in regulating spermatogenesis [127]. Furthermore, zebrafish with methimazole-induced hypothyroidism exhibited reduced germ cell differentiation, impaired meiosis, and a decreased number of spermatozoa in their testes, reinforcing the critical role of thyroid hormones in male fertility [128,129].

## 3. The Vasopressin/Oxytocin System

**Figure 1:**
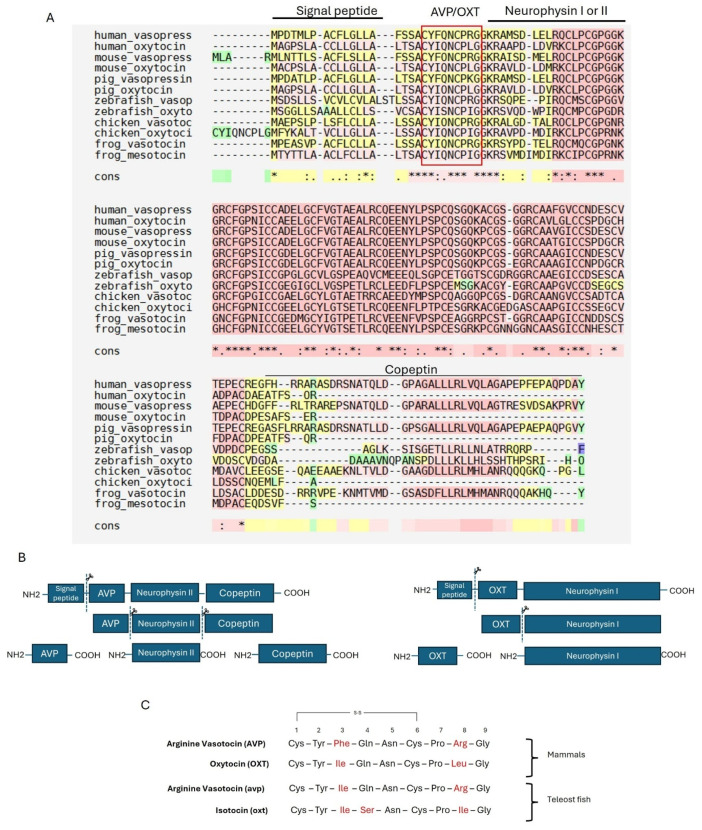
(**A**) Multiple sequence alignment (MSA) results generated by T-Coffee for the vasopressin and oxytocin family peptides across multiple vertebrate species: human (*Homo sapiens*), mouse (*Mus musculus*), pig (*Sus scrofa*), zebrafish (*Danio rerio*), chicken (*Gallus gallus*), and frog (*Xenopus laevis*). Sequences are aligned into different regions: signal peptide, active peptide (AVP/OXT), neurophysin I or II, and copeptin. Similar colors denote the level of similarity across specific regions. Asterisks (*) mark fully conserved residues, colons (:) and periods (.) indicate strong and weak similarity, respectively. Background colors indicate conservation: warmer colors (e.g., red) show higher conservation, cooler colors (e.g., green/blue) show lower conservation. (**B**) Schematic representation of the precursor proteins for AVP and OXT in mammals. Each precursor consists of a signal peptide, the nonapeptide hormone (AVP/OXT), a carrier protein (neurophysin I or II), and copeptin (in the case of AVP). The dashed lines indicate cleavage sites where the precursor is processed into its active components. (**C**) Alignment of the core nonapeptide sequences of vasopressin/oxytocin family neuropeptides in mammals and teleost fish. The conserved cysteine residues (Cys) at positions 1 and 6 form an intramolecular disulfide bridge (S–S). Key amino acid differences between the species are highlighted in red. NCBI accession numbers in the order of sequence appearance: NP_000481.2, AAA59977.1, AAC42027.1, EDL28279.1, NP_999117.1, NP_001161061.1, NP_840078.1, NP_840076.1, NP_990516.1, XP_040527589.1, XP_018098084.1, XP_018080922.1.

Arginine vasopressin (AVP) and oxytocin (OXT) are nonapeptides synthesized as part of the hypothalamic–neurohypophyseal system (HNS) and are involved in osmoregulation, metabolism, behavior, stress response, and reproduction [130,131,132]. The evolutionary lineages of AVP and OXT-like peptides evolved from a single ancestral arginine vasotocin (*avp*) gene after two rounds of whole genome duplication (2R WGD), which occurred during early vertebrate evolution [133,134]. These peptides are present and structurally well-conserved in all vertebrates (Figure 1A), with a nine-amino-acid composition and a disulfide bridge connecting the cysteines in positions 1 and 6 [134]. The teleost homologs of AVP and OXT are arginine vasotocin (AVP) and isotocin (OXT) [135,136]. Teleost vasotocin differs from mammalian vasopressin by a single amino acid in position 3 [131,134], and isotocin differs from mammalian OXT by two amino acids in positions 4 and 8 (Figure 1B) [131,134].

AVP and OXT are synthesized as larger precursor proteins by magnocellular neurons in the supraoptic nucleus (SON) and the paraventricular nucleus (PVN) of the hypothalamus. These precursors are stored in vesicles within the axon terminals of the posterior pituitary and released into circulation in response to various stimuli [131]. The active nonapeptide hormone is generated through cleavage by prohormone convertases before being secreted into the bloodstream [137]. The prohormones consist of a signal sequence, a highly conserved nonapeptide (AVP or OXT), an acidic carrier protein (neurophysin I or II), and a C-terminal glycoprotein (copeptin), which is uniquely present in the vasopressin prohormone (Figure 1B) [137]. Notably, the AVP/OXT nonapeptide core is the most conserved region; however, the neurophysin domain is also highly conserved, ensuring the stability and proper transport of the active hormone. In contrast, copeptin is less conserved among vasopressins, and its biological function in fish remains largely unknown [137]. However, in mammals, copeptin has recently been utilized as a biomarker for stress-induced cardiovascular disease detection in clinical settings [138]. Interestingly, our multiple sequence alignment (MSA) reveals the presence of copeptin in the zebrafish oxytocin prohormone (Figure 1A), although its sequence differs from that of copeptin in the AVP prohormone. This finding aligns with previous studies that identified copeptin in the oxytocin prohormone of other teleost fish, also using bioinformatic tools [139]. It has been proposed that copeptin may be related to the correct folding of vasopressin neurophysin II during precursor processing [139].

### The Receptors

The biological effects of arginine vasopressin and oxytocin are mediated by seven-pass transmembrane receptors that belong to the superfamily of G-protein-coupled receptors (GPCRs), a class of rhodopsin-like receptors. In mammals, three vasopressin receptors belonging to three different genes have been isolated and characterized: AVPR1A (V1a) and AVPR1B (V1B), which are expressed primarily in the central nervous system (CNS), and AVPR2 (V2), which is expressed in the periphery [140].

As a result of the teleost-specific whole genome duplication event, this group possesses two *avpr1a* (*avpr1aa* and *avpr1ab*), two *avpr2* (*avpr2aa* and *avpr2ab*), and *avpr2-like* (*avpr2l*) isoforms [141,142]. One single oxytocin receptor, termed OXTR, has been described in mammals, corresponding to two copies in teleosts, namely oxytocin receptor a (*oxtra*) and oxytocin receptor b (*oxtrb*) [142,143].

During evolution, the AVP and OXT receptor genes (*avpr* and *oxtr*, respectively) were subjected to lineage-specific losses following duplications (WGD and teleost-specific) [143]. For example, among the Actinopterygii (ray-finned) class, only the stickleback (family Gasterosteidae) genome has lost one copy of the oxytocin receptor (*oxtrb*) [142]. Furthermore, the *avpr1b* subtype is present in basal ray-finned fishes and tetrapods but was lost early in the evolution of elasmobranchs and teleosts [142,143]. The absence of *avpr1b* in elasmobranchs may be associated with their distinct osmoregulatory mechanism, which depends on urea retention to regulate osmotic balance. In contrast, species that lack this system—such as early-diverging ray-finned fishes and tetrapods—may have maintained *avpr1b* due to its functional importance in osmoregulation or other physiological roles subject to evolutionary pressures [143]. Additionally, the *avpr2b* receptor subtype is missing in several teleost species, including the Atlantic herring (*Clupea harengus*), zebrafish (*Danio rerio*), red-bellied piranha (*Pygocentrus nattereri*), electric eel (*Electrophorus electricus*), and striped catfish (*Pangasianodon hypophthalmus*). Overall, this suggests that varying evolutionary pressures have shaped receptor retention and loss in fish species, potentially leading to functional divergence or redundancy in these lineages.

The expression of AVP receptors is the highest in the brain, except for *avpr2l*, which is primarily expressed in the adrenal cortex and appears to be involved in mediating the stress response [141,144]. Tong et al. (2020) reported that in adult zebrafish, *avpr2ab* is mainly expressed in the kidney, while *avpr1ab* level is higher in the gills [145]. mRNA expression of vasotocin receptors was also detected through quantitative PCR (qPCR) in the testes of zebrafish. We previously analyzed the tissue-specific expression profiles of *avpr* paralogs in zebrafish, focusing on the brain and testis tissues [8]. As expected, all five *avpr* genes (*avpr1aa*, *avpr1ab*, *avpr2aa*, *avpr2ab*, and *avpr2l*) displayed the highest expression in the brain, with *avpr1aa*, *avpr1ab*, *avpr2aa*, and *avpr2l* also showing moderate to high expression in the testes.

All AVP and OXT receptors are coupled with G_q/11_, which uses diacylglycerol (DAG), inositol triphosphate (IP3), and Ca^2+^ as second messengers in their signal transduction pathways, except for AVPR2L, which is coupled with G_α_ and uses cAMP [144].

Functional and pharmacological studies on the receptor system showed that the AVPR1AB receptor has a strong preference for AVP over OXT in the white sucker (*Catostomus commersoni*) [146]. In contrast, the OXTRA receptor binds both nonapeptides but has an approximately three times higher affinity for OXT (80 nM) than AVP (300 nM) [147]. Recent studies in zebrafish have revealed that OXT effectively activates both OXT receptors (OXTRA and OXTRB) as well as the AVPR1AB receptor, with high potency (EC_50_ around 3 nM) [148]. In comparison, AVP exhibits the strongest activation of the AVPR1AB receptor (EC_50_ = 2.22 nM) while showing a slightly lower potency on the two OXT receptors [148]. These findings suggest a degree of cross-reactivity between the nonapeptides and their receptors in zebrafish, which is consistent with what has been demonstrated in mammalian species [149].

## 4. Autocrine/Paracrine Effect of Vasopressin and Oxytocin in the Gonads

### 4.1. In Mammals

Before discussing the roles of the nonapeptides arginine vasopressin and oxytocin in regulating gonadal function in fish, it would be beneficial to examine their functions in mammalian models. Recent studies in mammals are scarce, and most of the available information comes from studies conducted in the 1990s and early 2000s. In mammals, as in fish, OXT and AVP are synthesized both in the brain and peripheral tissues, including the testes and ovaries. Oxytocin production has been observed in Leydig and Sertoli cells across various mammalian species [150,151], whereas vasopressin production has been demonstrated in isolated Leydig cells from rat and mouse testes. Similarly, in the ovaries, both nonapeptides are produced in the granulosa cells [152,153].

The effects of OXT and AVP on ovarian function have been shown to vary across species, with mixed results. Oxytocin receptors are found in the Leydig and Sertoli cells of both human and macaque testes [154], and vasopressin receptors are present in the smooth muscle-like myoid cells of the seminiferous tubules [155]. In male mammals, oxytocin has been shown to increase fluid flow and sperm count in the rete testes of rams [156] and promote sperm transport to the epididymis in prepubertal rats [157]. Oxytocin treatment of rats for 28 days (long-term) induced a decrease in testosterone but an increase in dihydrotestosterone (DHT) plasma levels without affecting Leydig cell numbers, LH levels, or epididymal sperm counts [158]. While OXT knockout mice did not show significant differences in the testicular structure or germ cell development compared to wild-type mice [157], later studies demonstrated that oxytocin treatment increased spermatocyte and spermatid numbers, germ cell proliferation, and testosterone biosynthesis in prepubertal mice [159]. LH stimulates OXT production in Leydig cells, independent of testosterone levels [160]. OXT can, in turn, stimulate testosterone production in several species [159,161]. Overall, these studies highlight oxytocin’s regulatory role in male reproduction, suggesting its involvement in modulating the final stages of spermatogenesis, including sperm maturation, sperm movement, and hormone production across different mammalian species.

In terms of vasopressin’s effects, AVP was found to stimulate short-term (2 h) testosterone production in rat Leydig cells but inhibited it after prolonged exposure in the presence of hCG [160]. Similarly, AVP reduced LH-induced testosterone production, while OXT had no such effect [162]. In vitro studies also suggest that AVP impairs sperm function by reducing sperm motility, capacitation, and fertilization in mice [163]. AVP appeared to stimulate testosterone production in rat Leydig cells for up to 5 h, while in the presence of hCG, AVP inhibited testosterone production beyond 24 h of culture [164].

In females, oxytocin and vasopressin regulate ovarian functions, including follicular maturation, steroidogenesis, and ovulation. In pigs, OXT has been shown to modulate ovarian steroidogenesis by influencing progesterone secretion [165]. OXT also affects ovarian tissue contractility, contributing to oocyte release during ovulation, as demonstrated in marmoset monkeys [166]. AVP may play a role in follicular rupture and oocyte release, with evidence suggesting that it controls ovarian contractions, which may aid in oocyte expulsion during ovulation [167]. Overall, the nonapeptides oxytocin and vasopressin play critical yet complex roles in gonadal function across mammalian systems. Understanding their functions in mammals provides an important context for exploring their roles in fish gonadal biology.

### 4.2. In Fish

Research on the role of the vasotocin/isotocin system in gonadal functions in teleosts is not as extensive as in mammals. Limited information is available and varies across species, reproductive strategies, and methods of investigation. While much work has focused on the effects of nonapeptides in relation to reproductive behavior [6,168,169], their local functions in the gonads, particularly in males, remain poorly understood. Here, we provide an overview of what is currently known about the local functions of AVP and OXT in teleost fish. Circulating levels of isotocin in fish have been reported in the range of 0.1–0.7 pM for both males and females in round gobies (*Neogobius melanostomus*) [170], while circulating levels of vasotocin are in the range of 0.2–10 pM [130,170]. These low concentrations of circulating nonapeptides are unlikely to have a direct effect on the gonads. In this context, evidence has accumulated indicating that the gonads are a peripheral site of nonapeptide production. Although information on the autocrine or paracrine effects of AVP/OXT on the gonadal function remains scarce, we explored the most recent updates on this topic.

#### 4.2.1. Bioinformatic Approach

**Figure 2:**
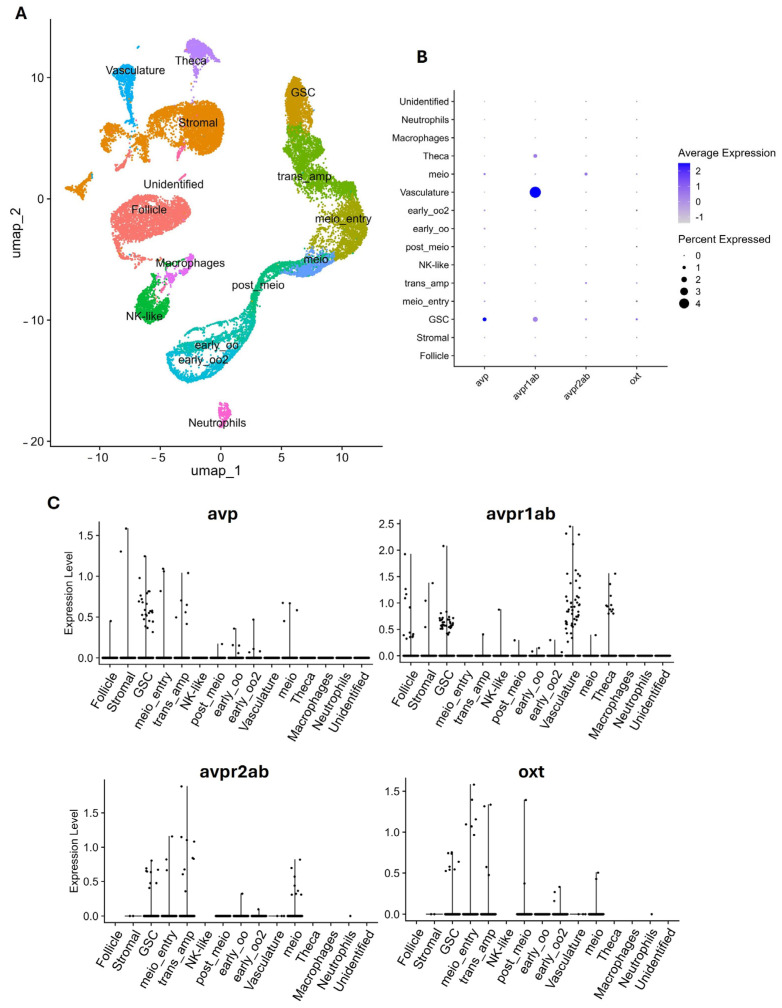
(**A**) UMAP representation of the cell populations in the adult ovaries of zebrafish. (**B**) Dot plot showing *avp*, *avpr1ab*, *avpr2ab*, and *oxt* expression levels across different cell clusters. (**C**) Violin plots representing gene expression. Each violin plot depicts the expression distribution of *avp*, *avpr1ab*, *avpr2ab*, and *oxt* across different cell types. Cell types include follicle cells, stromal cells, germline stem cells (GSCs), cells entering meiosis (meio_entry), transit-amplifying cells (trans_amp), natural killer-like cells (NK-like), post-meiotic germ cells (post_meio), early oocytes (early_oo and early_oo2), vasculature-associated cells, meiotic oocytes (meio), theca cells, macrophages, neutrophils, and a small group of unidentified cells.

**Figure 3:**
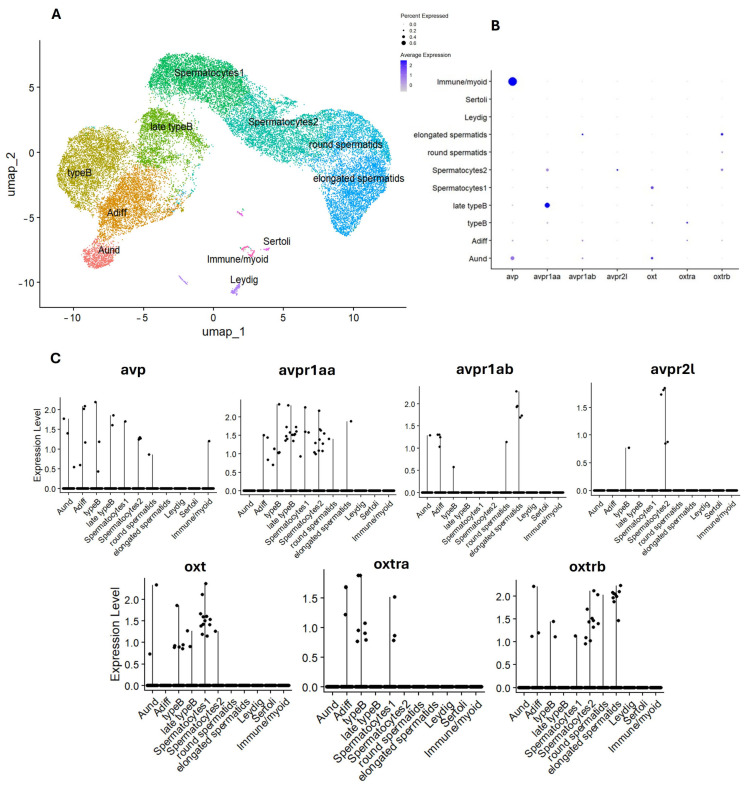
(**A**) UMAP representation of 10 different cell populations in adult zebrafish testes. (**B**) Dot plot showing expression levels of *avp*, *avpr1aa*, *avpr1ab*, *avpr2a*, *oxt*, *oxtra*, and *oxtrb* across different cell clusters. (**C**) Violin plots representing gene expression. Each violin plot depicts the expression distribution of avp, avp receptors, oxt, and oxt receptors across different cell types. Cell types include type A undifferentiated spermatogonia (Aund), type A differentiated spermatogonia (Adiff), early type B spermatogonia (typeB), late type B spermatogonia (late typeB), germ cells that enter meiosis I (Spermatocytes1), germ cells that enter meiosis II (Spermatocytes2), haploid cells before the final maturation (round spermatids), fully mature haploid cells (elongated spermatids), Leydig cells (Leydig), Sertoli cells (Sertoli), and a small group of immune and peritubular myoid cells (Immune/myoid).

Using publicly available single-cell RNA sequencing (scRNA-seq) datasets provided by Qian et al. (2022) (available at https://ngdc.cncb.ac.cn/gsa/browse/CRA003925 (accessed on 24 October 2024) and on GitHub (Git version 2.43.0) at https://github.com/gangcai/zebTestis (accessed on 10 April 2025)) [171] and Liu et al. (2022) (available at NCBI GEO https://www.ncbi.nlm.nih.gov/geo/query/acc.cgi?acc=GSE191137 (accessed on 11 April 2025) and on GitHub at https://github.com/yulongliu68/zeb_ov_ssRNAseq (accessed on 13 April 2025)) [172], we investigated the expression patterns of *avp*, *oxt*, and their receptors in the gonads of adult zebrafish (Figure 2 and Figure 3). The expression of the analyzed genes is extremely low and differentially expressed across germ and somatic cells in both testes and ovaries (Figure 2B and Figure 3B).

The results indicate that while both nonapeptides, *AVP* and *OXT*, are present in the ovaries, only two of the five AVP receptors, and none of the known OXT receptors, are detectable in this tissue. Notably, *avp* is enriched in both somatic and germ cells, with the highest level observed in the rapidly proliferating oogonia, which are identified here as transit-amplifying cells (trans_amp). Among the receptors detected, *avpr1ab* is mainly enriched in the vasculature cell cluster, which typically includes endothelial cells (lining blood vessels) and pericytes/smooth muscle cells (supporting vessel structure) [173], whereas *avpr2ab* is predominantly expressed in early meiotic oocytes (meio_entry). This expression profile of *avp* and its receptors suggests a role for the vasotocin system in promoting germ cell proliferation and facilitating the transition from oogonia to meiotic entry, potentially mediated through *avpr2ab*. In contrast, *oxt* expression appears restricted to the germline compartment, with enrichment specifically in the self-renewing germline stem cell (GSC) population (Figure 2 and Supplementary Table S1). Interestingly, neither of the known isotocin receptors (*oxtra* or *oxtrb*) was detected in the ovaries. However, this absence does not necessarily imply a lack of function for isotocin. The receptor expression may occur at levels below the detection threshold in this dataset or may be confined to specific developmental stages or physiological states not captured in the current analysis.

In the testes, *avp* expression is predominantly enriched in specific testicular cell populations, particularly in peritubular myoid and immune cells (Figure 3B). This is in line with previous research demonstrating the expression of *avp* prohormone in the testicular interstitial compartment in cichlid fish [6]. In contrast, *oxt* appears to reach its highest expression level in undifferentiated type A spermatogonia (Supplementary Table S2). This expression pattern in fish is notably different from that of mammals, where OXT is expressed in Sertoli cells [150,151]. This difference highlights a species-specific variation in the localization of OXT expression during gametogenesis. Regarding the nonapeptide receptors, it is noteworthy that they are present across all germ cells with peaks in expression levels, suggesting that each receptor subtype may play a stage-specific role during germ cell development (Figure 3C). *Avpr1aa* exhibits the highest expression level among other vasotocin receptor subtypes, with its expression primarily found in type B spermatogonia. In contrast, *avpr1ab* is more highly expressed in elongated spermatids, and *avpr2l* shows a higher expression in spermatocytes 2 (Supplementary Table S2). As for the isotocin receptors, *oxtrb* is the most abundant subtype and is predominantly expressed in elongated spermatids, while *oxtra* is mainly enriched in type B spermatogonia (Supplementary Table S2). Detailed methodology for the RNA-seq analysis is provided in Supplementary File S1.

#### 4.2.2. Ovaries: Local Production of AVP/OXT in the Ovaries and Role in Oogenesis

**Figure 4:**
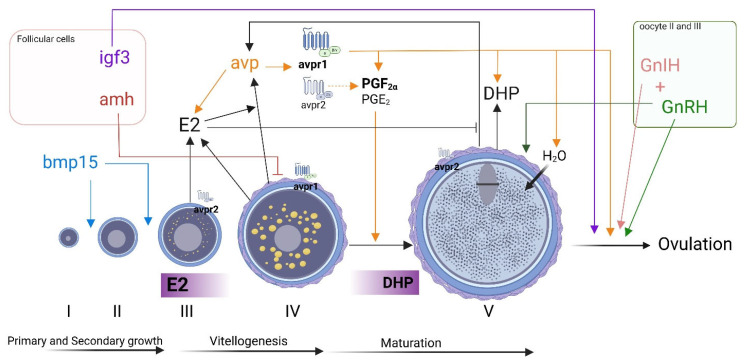
Schematic illustration of the factors involved in the autocrine/paracrine regulation of ovarian functions and oocyte development in fish, including vasotocin (avp). Oocyte classification follows the zebrafish nomenclature: I—primary-growth oocyte; II—secondary-growth oocyte; III—previtellogenic oocyte; IV—vitellogenesis; V—mature oocyte undergoing GVBD. Abbreviations: E2: estradiol-17β; DHP: 17α,20β-dihydroxy-4-pregnen-3-one; bmp15: bone morphogenic protein 15; amh: anti-Müllerian hormone; igf3: insulin-like growth factor 3; avp: arginine-vasotocin; avpr1: vasotocin receptor type 1; avpr2: vasotocin receptor type 2; PGF_2α_: prostaglandin F2α; PGE_2_: prostaglandin E_2_; GnIH: gonadotropin-inhibitory hormone; GnRH: gonadotropin-releasing hormone; H_2_O: water. Each factor is depicted in a unique color to facilitate clear identification.

A series of studies conducted by the same research group has consistently investigated the effects of AVP on the ovaries of Asian stinging catfish (*Heteropneustes fossilis)*, providing valuable insights into its role in ovarian function and regulation. AVP was first detected via high-performance liquid chromatography (HPLC) and later localized through immunocytochemistry in both the theca and granulosa cells of *H. fossilis* oocytes [174]. Similarly, in the seabream (*Sparus aurata*), *avp* expression was confirmed in the granulosa and theca cells associated with vitellogenic oocytes, oocytes in maturation, and oocytes in the hydration stage. However, nonapeptides were absent in the follicular cells surrounding the primary oocytes [175]. AVP was found to induce physiological ovarian changes in catfish, including increased oocyte water content, diameter, volume, osmolality, Na^+^/K^+^ ATPase activity, intracellular cation concentrations, GVBD, and ovulation [7]. The same group demonstrated that AVP, more than hCG, stimulates the synthesis of prostaglandins (PGF_2α_ and PGE), primarily acting through the V1-type receptor, inducing GVBD in post-vitellogenic oocytes (Figure 4) [176]. In this context, the synthesis of prostaglandins appears to be essential for AVP to fully promote oocyte maturation and ovulation. AVP receptors have also been localized in the ovaries across different species, including Asian stinging catfish (*H. fossilis*), bluehead wrasse (*T. bifasciatum*), and seabream (*S. aurata*) [175,177,178]. In catfish, during the pre-spawning phase, V1A-type receptors (AVPR1AA and AVPR1AB) are found in the follicular layer but not in the oocytes. At the same time, V2A (AVPR2AA) localizes to the oocyte membrane but is absent in follicular cells [177]. The presence of three distinct AVP receptor types in the catfish ovary suggests a regulatory role for AVP in ovarian functions, with their differential distribution indicating that each receptor subtype may mediate specific functions through distinct signaling pathways. More recently, Ferré et al. (2023) further demonstrated in the seabream that the *avpr1aa* and *avpr2aa* subtypes are expressed in isolated follicles with AVPR1AA restricted to vitellogenic oocytes while AVPR2AA present in pre- and post-vitellogenic oocytes and follicular cells [175]. These findings suggest that the vasopressinergic system may be involved in the final step of oogenesis and that AVP may regulate oocyte hydration by mediating the trafficking of the aquaporins AQP1AB1/AQP1AB2 to maximize water influx [175]. The known effects of vasotocin on the fish ovary are summarized in Figure 4.

#### 4.2.3. Interaction of AVP/OXT with Ovarian Factors

In catfish (*H. fossilis*), AVP promotes GVBD and final oocyte maturation by inducing a steroidogenic shift in post-vitellogenic follicles, inhibiting E2 but increasing maturation-inducing steroids and progestins [7]. In line with this study, evidence suggests that in seabream, vasotocin and isotocin stimulate E2 and progestins, depending on the dose, follicular stage, season, and incubation time. Low doses of AVP up to 100 nM stimulated E2 in previtellogenic oocytes while inducing progestins in post-vitellogenic oocytes [175]. Female zebrafish with *avp* knockout exhibited an overall decrease in oocytes, with a reduced percentage of oocytes in stage I and a higher percentage of oocytes in the maturation stage (stage V) compared to wild-type ovaries [13]. The accumulation of mature oocytes in the ovaries of *avp^−/−^* mutants underlines a failure in oocyte ovulation, which strongly indicates a role for AVP in egg release [13]. In the Nile tilapia (*O. niloticus*), isotocin has been shown to influence the expression of genes related to gonadal development and function, such as gonadotropin receptor genes (*fshr* and *lhcgr*). In this study, isotocin injections caused a peak in the production of E2 and T after 24 h in females and males, respectively [179]. Together, these findings highlight the role of AVP and OXT in fish ovarian function, influencing steroidogenesis, oocyte maturation, and ovulation through species-specific and stage-dependent mechanisms.

#### 4.2.4. Regulation of AVP/OXT Production in the Ovaries

Incubation with different doses of E2 (1, 10, and 100 ng/mL) significantly enhanced ovarian AVP production in the previtellogenic catfish ovaries in vitro in a dose- and duration-dependent manner. On the other hand, post-vitellogenic ovaries incubated with E2 showed a decrease in vasotocin production [9]. The same study also demonstrated that follicles incubated with hCG, progesterone (P4), or DHP increased vasotocin production, with the most significant effect observed after 16 h of incubation [9]. In Asian stinging catfish, injections with hCG (100 IU hCG/fish) stimulated AVP production in the brain, ovaries, and plasma, with tissue-specific responses. Interestingly, AVP release in the brain peaked at 8 h post-injection while reaching its highest concentration in the plasma and ovaries 16 h after hCG administration, suggesting that the rate of vasotocin release and transport into the circulation was higher than hypothalamic synthesis [180]. This study found that ovariectomized catfish, which had reduced plasma E2 levels, also showed lower AVP levels in both the brain and plasma. However, when given a low dose of E2 replacement, AVP levels were restored [9]. Together, these results indicate that both hCG and ovarian estrogen can influence ovarian AVP secretion. Since E2 altered ovarian AVP release in a follicular stage-dependent manner, E2’s effects on brain AVP follow a similar pattern. However, in the brain, E2 likely functions as an endocrine feedback regulator. At the same time, in the ovaries, it acts as an autocrine or paracrine factor, stimulating AVP production by the granulosa cells [9,180]. A schematic representation of the effect of AVP in the ovaries of fish is illustrated in Figure 4.

### 4.3. Testes: Local Production of AVP/OXTin the Testes and Role in Spermatogenesis

Much less information is available on the role of nonapeptides in males. Studies on Asian stinging catfish, *H. fossilis*, have demonstrated the testis localization of all three vasotocin receptors. Specifically, V1A2 and V2A were abundantly expressed in the interstitial compartment and the seminiferous epithelium, whereas V1A1 had an overall lower expression [177]. Vasotocin preprohormone was also detected by RT-qPCR in the testicular interstitial compartment of cichlid fish *Cichlasoma dimerus* [6]. Consistent with previous studies, we confirmed the expression of mRNA transcripts of *avp* and the five vasotocin receptor subtypes in the testes of adult zebrafish [8].

**Figure 5:**
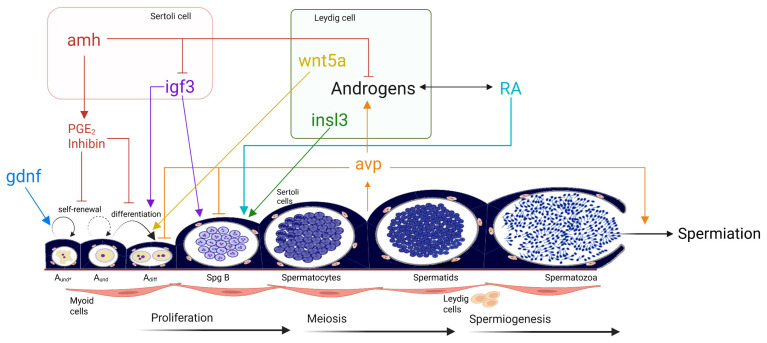
Schematic representation of the local role of vasotocin (avp) on zebrafish spermatogenesis. Germ cell development includes undifferentiated spermatogonia or stem cells (Aund*, the asterisk marks Aund stem/progenitor cells), type A undifferentiated non-stem cells (Aund), type A differentiated cells (Adiff), type B cells (SpgB), spermatocytes, spermatids, and spermatozoa. Abbreviations: gdnf: glial cell line-derived neurotrophic factor; amh: anti-Müllerian hormone; igf3: insulin-like growth factor 3; inls3: insulin-like peptide 3; avp: arginine-vasotocin; PGE_2_: prostaglandin E_2_; Wnt5a: wingless-type MMTV integration site family member 5A; RA: retinoic acid. Each factor is depicted in a unique color to facilitate clear identification.

A study on adult zebrafish in vivo revealed that both nonapeptides are crucial for male reproductive success, with vasotocin specifically affecting courtship behavior. Interestingly, the injection of vasotocin and isotocin receptor antagonists (Manning compound and L-368,899 hydrochloride, respectively) inhibited male courtship behavior, inducing a synergistic effect that indicates a complex interplay between these systems [12]. In addition, this study found no changes in the levels of T and 11-KT production upon treatment with nonapeptide receptor antagonists [12]. In contrast to these findings, adult zebrafish testes treated ex vivo with 10 nM AVP showed elevated levels of 11-KT and an increased production of spermatozoa. In contrast, the proliferation of type B spermatogonia was inhibited. Together, these data suggest that while AVP stimulates short-term androgen-dependent spermiogenesis, its prolonged actions may lead to diminished spermatogenesis through decreased spermatogonia proliferation (Figure 5) [8]. It is important to underline that, although FSH and LH are not explicitly shown in Figure 4 and Figure 5, they are essential factors, stimulating spermatogenesis and the overall reproductive processes.

#### Interaction of AVP/OXT with Testicular Factors

One of the first studies investigating the effects of AVP and OXT on reproduction was conducted in rainbow trout (*Oncorhynchus mykiss*), demonstrating that both AVP and OXT stimulated testosterone production in an immature testis culture (ex vivo). At the same time, there was no response in mature testes [181]. The authors observed a maximal response at a concentration of 10 nM, with isotocin affecting testosterone production to a lesser degree than vasotocin [181]. Isotocin has been demonstrated to have similar effects to vasotocin when administered at higher concentrations in several species [4,182]. Similarly, exposure of testicular fragments to vasotocin induced a dose-dependent stimulation of androgen production in cichlid fish *Cichlasoma dimerus* [6]. The increased level of androgens, particularly 11-KT, was associated with increased aggressive behavior linked to social hierarchy formation and territorial status. Overall, the results suggest that AVP likely influences these behaviors at a central level as a neuromodulator while also acting peripherally as a pituitary hormone-releasing factor that stimulates androgen synthesis in the testes [6].

## 5. Knowledge Gaps and Future Directions

Despite the growing body of evidence supporting the role of vasotocin and isotocin in fish gonadal function, significant gaps remain in our understanding of their autocrine and paracrine actions in both the ovary and testis.

In teleost ovaries, AVP receptors have been detected in follicular cells, theca cells, granulosa cells, and oocytes. However, their specific physiological roles in each cell type remain largely unexplored. In addition, the functional significance of receptor subtype distribution is unclear—do different receptors mediate distinct processes such as steroidogenesis, hydration, or ovulation? Information on how AVP receptor expression changes throughout the ovarian cycle, particularly in early versus late oogenesis, is also limited. The species-specific and stage-dependent effects of these nonapeptides on ovarian steroidogenesis, oocyte maturation, and ovulation highlight the complexity of their regulatory mechanisms, which may also involve additional environmental or molecular factors that remain to be explored. Experimental evidence provided by multiple research groups [175,183] demonstrates vasotocin’s role in the regulation of oocyte hydration through aquaporin trafficking, a crucial process for preparing the oocyte for ovulation. However, further studies are needed to fully elucidate the underlying molecular mechanisms and regulatory pathways involved, as well as their presence in other species. Similarly, the regulation of ovarian AVP production by E2 and gonadotropins [7] in Asian stinging catfish suggests a feedback mechanism that integrates hypothalamic, pituitary, and ovarian signals. However, the molecular basis of this interaction remains to be fully characterized across multiple fish species.

The expression of vasotocin and its receptors in the testes of zebrafish (*Danio rerio*), Asian stinging catfish (*Heteropneustes fossilis*), and cichlid fish (*Cichlasoma dimerus*) indicates a potential role in spermatogenesis, but the functional relevance of this system in regulating germ cell progression, steroidogenesis, and sperm maturation is not well-understood. The differential expression of AVP receptors across various germ and somatic cells suggests cell type-specific functions, yet the downstream signaling pathways mediating these effects remain unidentified. Likewise, the near-complete lack of knowledge regarding the regulation of testicular AVP and OXT production significantly limits our understanding of their role in male reproductive physiology. The available data on this subject are limited to mammalian models, as previously discussed (see Section 4.1). Future research should focus on elucidating the molecular mechanisms underlying nonapeptides’ action in the testes, utilizing knockout models and -omics approaches such as single-cell RNAseq. Although recent findings in zebrafish have suggested a dual role for AVP in promoting short-term androgen-dependent spermiogenesis and potentially inhibiting earlier stages of spermatogenesis [8], further studies are needed to fully elucidate these mechanisms and provide a complete picture of the physiological function of AVP and its receptors. For example, employing receptor antagonists, as well as co-administration of nonapeptides with known regulatory factors such as gonadotropins, will be essential for distinguishing between the direct and indirect effects on spermatogenesis and elucidating the functional role of AVP and OXT within the broader framework of the complex and multifactorial regulation of testicular function.

## 6. Conclusions

**Figure 6:**
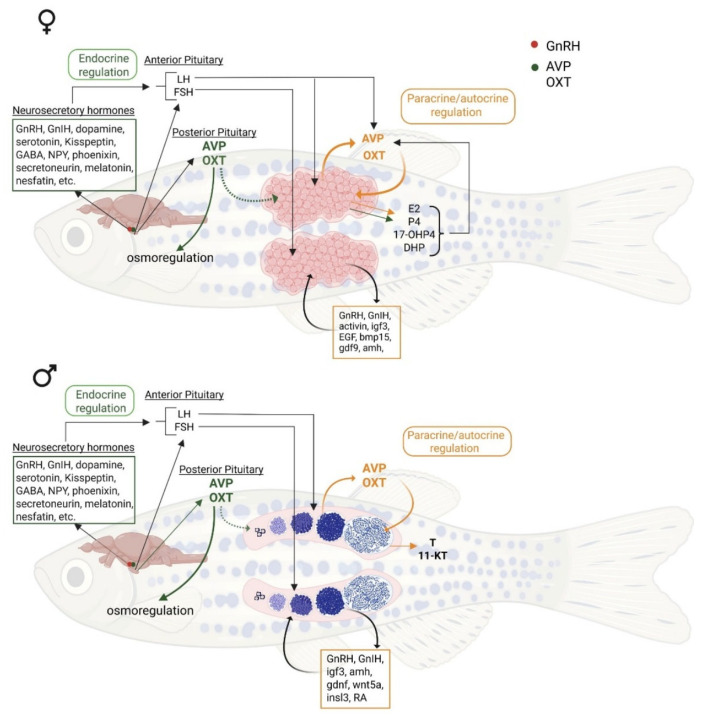
Schematic overview of neuroendocrine and paracrine/autocrine regulation of reproduction in female (**top**) and male (**bottom**) fish. The diagram illustrates the integration of neurosecretory hormones and classical endocrine signals from the anterior and posterior pituitary, highlighting the roles of AVP and OXT in both central and local (paracrine/autocrine) regulation. Key reproductive hormones and factors are shown, including gonadotropin-releasing hormone (GnRH), gonadotropin-inhibitory hormone (GnIH), gamma-aminobutyric acid (GABA), neuropeptide Y (NPY), luteinizing hormone (LH), follicle-stimulating hormone (FSH), estradiol-17β (E2), progesterone (P4), 17α-hydroxyprogesterone (17-OHP4), 17α,20β-dihydroxy-4-pregnen-3-one (DHP), testosterone (T), 11-ketotestosterone (11-KT), insulin-like growth factor 3 (igf3), epidermal growth factor (EGF), bone morphogenetic protein 15 (bmp15), growth differentiation factor 9 (GDF9), anti-Müllerian hormone (amh), glial cell line-derived neurotrophic factor (gdnf), Wnt family member 5A (wnt5a), insulin-like peptide 3 (insl3), and retinoic acid (RA).

While the neurohypophyseal nonapeptides, AVP and OXT, have been classically associated with the neuroendocrine regulation of osmoregulation, blood pressure, lactation, and parturition in mammals, recent research suggests that they also contribute to the local control of gametogenesis. Several studies have provided insights into how AVP and OXT impact germ cell development, steroidogenesis, and gamete maturation. In fish, AVT has been shown to enhance the proliferation and differentiation of early spermatogonial germ cells, as well as the late stages of spermiogenesis in a different way. It is now clear that while pituitary gonadotropins are the primary drivers of spermatogenesis and oogenesis, several peripheral and gonadal hormones contribute to the multifactorial control of gametogenesis in fish and other vertebrate species. The role of gonadal peptides, including AVP/OXT, has been extensively investigated by numerous researchers, and there is increasing evidence that paracrine/autocrine control of gametogenesis is crucial for ensuring the synchronous development of eggs and sperm, as well as ovulation, under appropriate environmental and physiological stimuli. Figure 6 (below) provides a summary of these neuroendocrine and paracrine/autocrine interactions in both female and male fish. Here, we highlighted the central and local actions of AVP and OXT, their integration with classical endocrine signals (such as LH and FSH), and their downstream effects on gametogenesis and steroid production. This integrative representation underscores the complexity of the reproductive process in fishes and other vertebrate species.

## Data Availability

No new data were created or analyzed in this study.

## Supplementary Materials

The following supporting information can be downloaded at https://www.mdpi.com/article/10.3390/cells14141061/s1, File S1 provides a detailed methodology for single-cell RNA-seq data processing and analysis. This file includes preprocessing, quality control, normalization, clustering, and gene expression analysis of zebrafish testis and ovary datasets. Table S1 includes expression levels of avp, oxt, and their receptors detected across cell populations in the ovary identified by single-cell RNAseq. Table S2 provides expression levels of avp, oxt, and their receptors (avpr1aa, avpr1ab, avpr2l, oxtra, oxtrb) in different testicular cell populations identified by single-cell RNAseq.
