# Supplementary material for: The Role of Neurohypophysial Hormones in the Endocrine and Paracrine Control of Gametogenesis in Fish
Source: Cells. 2025 Jul 10;14(14):1061. doi: 10.3390/cells14141061 (PMC12293452; doi:10.3390/cells14141061)
Supplement: Supplementary file 1 [file cells-14-01061-s001.zip › cells-3703851-supplementary.pdf]

Supplementary Materials

Supplementary File S1. Detailed methods for single-cell RNA-seq data processing and analysis

S1. Testis Dataset Analysis

We analyzed a publicly available single-cell RNA sequencing (scRNA-seq) dataset of adult zebrafish testis generated by Qian et al. (2022), accessible via the Genome Sequence Archive (https://ngdc.cncb.ac.cn/gsa/browse/CRA003925). Raw sequencing reads were processed using Cell Ranger v9.0.1 (10x Genomics) and aligned to the zebrafish reference genome GRCz11. Downstream analysis was performed in Seurat v5 within R v4.2.2.

Quality control was applied as follows: cells with ≥200 detected genes, cells with <5% mitochondrial gene content, and cells with >200 unique molecular identifiers (UMIs).

Data were log-normalized with a scale factor of 10,000. We identified the top 2,000 highly variable genes and scaled the data while regressing out mitochondrial gene expression.

Dimensionality reduction was performed using Principal Component Analysis (PCA) on the top 30 principal components. A shared nearest-neighbor graph was constructed, and clusters were identified using a resolution of 0.3. Clusters were visualized using UMAP and labeled with numeric cluster IDs, which were assigned to biologically meaningful cell types based on gene expression patterns.

Differential gene expression analysis was performed using the FindMarkers() function (Wilcoxon rank-sum test; log fold-change threshold = 0.25; adjusted p-value < 0.05). The top 20 marker genes per cluster were selected and sorted by log fold-change.

Expression of avp, avpr1aa, avpr1ab, avpr2l, oxt, oxtra, and oxtrb was analyzed using dot plots and violin plots. The spatial distribution of expressing cells was assessed in the UMAP projection. Avpr2aa and avpr2ab were not detected, likely due to low expression levels leading to filtering during quality control.

S2. Ovary Dataset Analysis

Three scRNA-seq datasets from 40 days post-fertilization (dpf) zebrafish ovaries were analyzed. These datasets were generated and deposited by Liu et al. (2022) and are available at: https://www.ncbi.nlm.nih.gov/geo/query/acc.cgi?acc=GSE191137.

Datasets:

- zx1_40gc: germ cell–rich sample
- zx2_40ov and zx4_40ov: somatic cell–enriched samples

Each dataset was preprocessed individually as described in Liu et al. (2022) and saved as a Seurat object. They were then merged into a single object using Seurat's merge() function, with sample-specific prefixes assigned to cell barcodes for traceability. Normalization and correction for technical variation were performed using SCTransform. Dimensionality reduction was carried out with PCA (top 30 components), followed by UMAP for visualization. A shared nearest-neighbor graph was built from PCA results, and clustering was done using the Louvain algorithm with a resolution of 0.5. Clusters were annotated based on UMAP projection and reference to Liu et al. (2022). Neuropeptide and receptor gene expression (*avp, avpr1aa, avpr1ab, avpr2aa, avpr2ab, avpr2, oxt, oxtr*) was assessed. Only genes detected in the dataset were retained for downstream visualization using UMAPs, dot plots, and violin plots.

Avpr1aa and *oxtr* were not detected, possibly due to low expression levels or lack of expression under the experimental conditions.

Table S1. Expression levels of the nonapeptides vasotocin (*avp*) and isotocin (*oxt*) and the receptors detected (*avpr1ab, avpr2ab*) across the cell populations in the ovary of zebrafish, identified by single-cell RNAseq. Expression values represent normalized counts. Bold values indicate the highest expression for each transcript. – represents zero expression.

Ovarian Cell Population	avp	avpr1ab	avpr2a	oxt
**GSC**	0.001907	-	0.002167	**0.007753**
**Trans_amp**	**0.011622**	0.018230	0.005314	0.003576
**Meio_entry**	0.003413	-	**0.007561**	0.000919
**meio**	-	-	0.001667	0.004294
**Post_meio**	0.000150	0.000277	-	0.002453
**Early.oo**	0.000606	0.000066	0.000092	0.000360
**Early.oo.2**	0.004412	0.000464	0.004984	0.000519
**Early.oo.3**	0.000574	0.000326	0.000503	-
**Early.oo.4**	0.001934	0.001120	-	0.001545
**Follicle**	0.000304	0.001420	-	-
**Stromal**	0.002286	0.002346	-	-
**Stromal.fgf24**	-	0.025030	-	-
**Theca**	-	0.012380	-	-
**Vasculature**	-	**0.045344**	-	-
**NK.like**	-	0.000655	-	-
**Macrophages**	-	-	-	-
**Neutrophils**	-	-	-	-
**Unidentified**	-	-	-	-

Table S2. Expression levels of vasotocin (avp), isotocin (oxt), and their receptors (*avpr1aa, avpr1ab, avpr2l, oxtra, oxtrb*) in different testicular cell populations identified by single-cell RNAseq. Expression values represent normalized counts. Bold values indicate the highest expression for each receptor. – represents zero expression.

Testicular Cell Population	avp	avpr1aa	avpr1ab	avpr2l	oxt	oxtra	oxtrb
**spermatocytes1**	0.000865	0.003479	-	-	0.009744	0.001183	0.000403
**spermatocytes2**	0.001524	0.007077	-	**0.003576**	0.000507	-	0.006605
**Type B**	0.002193	0.003676	0.000157	0.000236	0.002603	**0.003744**	0.001075
**elongated spermatids**	-	0.001204	**0.006457**	-	-	-	**0.01239**
**Adiff**	0.00431	0.00208	0.002489	-	-	0.002884	0.003238
**late type B**	0.003242	**0.018184**	-	-	0.001391	-	-
**round spermatids**	0.000699	0.001565	0.001087	-	-	-	0.00495
**Aund**	0.009302	-	0.002812	-	**0.011229**	-	-
**Leydig**	-	-	-	-	-	-	-
**Immune/myoid**	**0.01822**	-	-	-	-	-	-
**Sertoli**	-	-	-	-	-	-	-
